# Canine Visceral Leishmaniasis; A Seroepidemiological Survey in Jiroft District, Southern Kerman Province, Southeastern Iran in 2015

**Published:** 2018

**Authors:** Mohammad Javad ABBASZADEH AFSHAR, Iraj SHARIFI, Mehdi BAMOROVAT, Mehdi MOHEBALI, Mohammad Saleh BAHREINI, Afsaneh NADERI

**Affiliations:** 1. Dept. of Medical Parasitology and Mycology, School of Public Health, Tehran University of Medical Sciences, Tehran, Iran; 2. Research Center of Tropical and Infectious Diseases, Kerman University of Medical Sciences, Kerman, Iran; 3. Leishmaniasis Research Center, School of Medicine, Kerman University of Medical Sciences, Kerman, Iran; 4. Center for Research of Endemic Parasites of Iran (CREPI), Tehran University of Medical Sciences, Tehran, Iran; 5. Dept. of Medical Parasitology and Mycology, School of Medicine, Shiraz University of Medical Sciences, Shiraz, Iran; 6. Iranian Social Security Organization, Jiroft, Kerman, Iran

**Keywords:** Canine visceral leishmaniasis, Direct agglutination test (DAT), Iran

## Abstract

**Background:**

Domestic dogs have been implicated as the main reservoir host of Mediterranean type of visceral leishmaniasis (VL) that is endemic in some parts of Iran. This study was performed about role of dogs in canine VL (CVL) epidemiology in Jiroft District, south of Kerman Province, southeastern Iran.

**Methods:**

Totally, 165 dogs including 100 stray and 65 sheepdogs were randomly selected. After complete clinical examination blood sample was taken from each dog. All the collected samples were examined following the serum separation by direct agglutination test (DAT) for detection of anti-*Leishmania infantum* antibodies. The titers of ≥1:320 were defined as positive.

**Results:**

Overall, of 165 serum samples, 13 samples (7.9%) were positive by DAT at titers of ≥1:320. The seroprevalence was 11% among the stray dogs and 3% among the sheepdogs. There was no significant difference between stray and sheepdogs in CVL infection. The highest seroprevalence rate (14.3%) was found in seven-year old dogs.

**Conclusion:**

The present finding indicates the role of stray and sheepdogs in CVL epidemiology in this area. Further investigations are needed to evaluate the status of VL infection in human subjects in this area.

## Introduction

Canine visceral leishmaniasis (CVL) caused by *Leishmania infantum* is endemic in Iran and transmitted to human by the bite of female sandflies. This disease is not only a veterinary problem but also is a serious public health concern in endemic countries. Therefore, rapid detection of CVL is highly important for controlling human visceral leishmaniasis (HVL) (1, 2).

Domestic dogs as most important reservoir hosts are considered as an important risk factor for human infection in the endemic areas of the disease in Iran (3). Clinical manifestations in dogs are various, such as weight loss, cachexia, ocular lesions, lymphadenopathy, dermatitis, alopecia, cutaneous ulcerations, anorexia, epistaxis, anemia and diarrhea (4, 5).

In the present study, DAT was used as a serodiagnostic tool, since it is a valid, cost effective, sensitive/specific and user-friendly test (6). The current investigation was performed to determine the seroprevalence of CVL among stray and sheepdogs in Jiroft district, south of Kerman Province to evaluate the role of dogs as the potential reservoir of HVL. The reason for choosing this place was the previous study, which reported high prevalence of HVL from the study area (7).

## Methods

### Study area

**Fig. 1:**
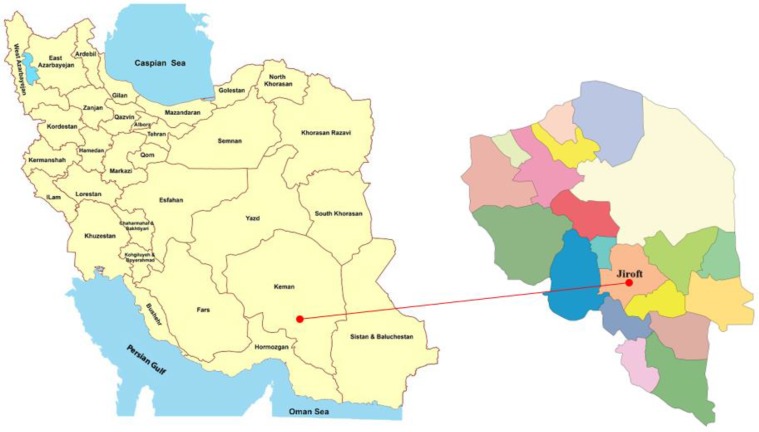
Situation of Kerman Province in Iran and location of study area in Kerman Province, Iran

Jiroft district with 277,748 populations is located approximately in south of Kerman Province (Fig.1). The study area has three different climate zones: cold, warm, and moderate. About 49% and 2% of district's population have rural and nomadic lifestyle, respectively: https://en.wikipedia.org/wiki/Jiroft_County).

### Ethical consideration

The owners of sheepdogs participating in this study signed the consent form before sampling. The study was approved by the Ethics Committee of the university.

### Blood sampling

This cross-sectional study was carried out as descriptive survey from May to August 2015 among stray and sheepdogs in Jiroft district.

Blood samples (3–5 ml) were taken from heart of 100 stray dogs that were sacrifice by Jiroft municipality office in order to control of stray dogs' population in the study area and not for use in this study. In addition, 65 sheep dogs were selected randomly and blood samples were taken from cephalic vein of them. A check-list including demographic characteristics (age, gender and lifestyle) and clinical status was completed for each dog after complete clinical examination. Teeth examination method was used to determine the approximate age of stray dogs and sheepdogs’ age was determined by interviewing dog owners. The blood samples were centrifuged at 800 g for 5 to 10 min and sera were separated and stored at −20 °C.

### DAT

The serum samples were tested by DATs (8). The plasma was diluted 1:80 for preliminary screening and the positive sample were serially diluted up to end to obtain the real titer for IgG antibody titration. The cutoff of DAT for CVL was considered 1:320 titers (8, 9). The highest titers at which agglutination was still visible was reported.

### Data analysis

A Chi-square test was used to determine significant differences between proportions. Analyses were performed using SPSS software version 18, with a probability (*P*) value of <0.05 as statistically significant level.

## Results

Sera samples were taken from 165 dogs consisting of 100 (60.6%) stray and 65 (39.4%) sheepdogs. The seroprevalence rate (SPR) in titers 1:320 and above was 7.9%. Eleven dogs out of 100 stray dogs (11%) and 2 dogs out of 65 sheepdogs (3%) were seropositive. There was no significant differences of *Leishmania* infection between these two groups of dogs *(P=*0.065*)*. The highest seroprevalence rate (14.3%) was found in seven-year old dogs. On the other hand, there was a significant difference between CVL infection and age groups *(P=*0.015*)*.

Nine out of 13 seropositive dogs showed clinical manifestation including skin lesions, weight loss and cachexia. No statically significant difference was found between *Leishmania* infection and clinical signs (*P=*0.110) (Table 1).

**Table 1:** *Leishmania infantum* infection among studied dogs regarding to Lifestyle, clinical status and age group in Jiroft district, Kerman province, Iran, 2015

***Parameter***	***Dogs No. (%)***	***DAT positive (≥1:320)***	
		**No.**	**Seroprevalence (%)**
**Lifestyle**			
Stray	100 (60.6)	11	11
Sheepdog	65 (39.4)	2	3
**Clinical status**			
Symptomatic	9 (5.5)	9	100
Non-symptomatic	156 (94.5)	4	2.5
**Age group(yr)**			
≤3	55 (33.3)	1	1.8
4–7	54 (32.7)	4	7.4
7<	56 (33.9)	8	14.3
**Total**	165 (100)	13	7.9

## Discussion

In the past decades, over 100 cases of HVL have been reported and passively registered in Kerman Province (7,11). Approximately one-third of cases were registered from the nomadic tribes of Jiroft district. The tribe's people in south of Kerman province (Soleimani and Jebalbarezi tribes) travel with their herds and sheepdogs each year from the Summer highland quarters in Baft district to Winter quarters, to lower (and warmer) lands in Jiroft district. It seems that tribe's dogs, constitute the main reservoirs for the infection in the nomadic tribes of south of Kerman province.

In accordance with our results, seroprevalence of CVL in tribal areas in south of Kerman province was determined to be 7.9% using the cut-off value of ≥1:320. Different seroprevalence rates of *L. infantum* infection among dogs have been previously reported in several parts of Iran. Based on a survey in 2014 in Kerman Province seroprevalence of CVL was determined as 15.4% by ELISA (10).

Mohebali et al. reported the seroprevalence rate of 4.4% among dogs in the South-west regions (3). In addition, the highest seroprevalences rate of CVL (17.4%) has been documented in Meshkin-Shahr District, Ardabil province (12). In another study, 7 out of 30 domestic dogs (23%) showed anti-*L. infantum* antibodies at titers ≥1:320 in Baft district, Kerman Province (13). In addition, Asgari et al. demonstrated a rate of 6.25% of *L. infantum* seroprevalence among Qashqaei tribe dogs in Fars province (14).

The infection rate of *L. infantum*, among stray and sheepdogs, by IFA, was 11.7% and 4.3%, in Khorasan Razavi (15). The prevalence of the infection in stray and sheepdogs in our survey was 11% and 3%, respectively. Although no statistical differences were found between CVL infection and dog's lifestyle but the seroprevalence rate was higher in stray dogs compared to sheepdogs. This difference is probably associated with the bite of sandflies which the stray dogs are exposed it, all day long.

In our survey, dogs of ≥7 years of age showed the highest seroprevalence rate (14.3%) and there was significant difference between dog's age and CVL infection. In 2008 a seroepidemiological study on 384 serum samples of owned dogs in Northwest of Iran showed the highest seroprevalence rate of *L. infantum* infection in dogs with 8 years and older (12). Anti-*L. infantum* antibodies increased with age of the dogs (12,16). This finding is consistent with the results obtained in this study.

In the present study, 9 out of 13 seropositive dogs (69%) showed clinical signs. In Meshkin-Shahr district, only 25.4% of seropositive dogs exhibited clinical signs (12). A survey in Fars province indicates four of the six seropositive dogs (67%) were asymptomatic (17). The dogs with no clinical symptoms are able to transmit VL to humans similar to the dogs with clinical symptoms. Therefore, this issue is important in relation to epidemiological aspects and the transmission of VL to humans. High numbers of infected dogs lacking clinical signs may confer protective immunity especially in older dogs as they get more chance of exposure to *Leishmania* parasites.

## Conclusion

The present finding indicates the role of stray and sheepdogs in CVL epidemiology in this area. Such dogs could be potential source of infection for transmission of visceral infection to humans. Since the nomadic life involves migration through south and north back and forth within the province, the disease has high potential dynamic of infection transmission to humans through the migration route.
